# A multi-centred randomised trial of radical surgery versus adjuvant chemoradiotherapy after local excision for early rectal cancer

**DOI:** 10.1186/s12885-016-2557-x

**Published:** 2016-07-21

**Authors:** W. A. A. Borstlap, P. J. Tanis, T. W. A. Koedam, C. A. M. Marijnen, C. Cunningham, E. Dekker, M. E. van Leerdam, G. Meijer, N. van Grieken, I. D. Nagtegaal, C. J. A. Punt, M. G. W. Dijkgraaf, J. H. De Wilt, G. Beets, E. J. de Graaf, A. A. W van Geloven, M. F. Gerhards, H. L. van Westreenen, A. W. H. van de Ven, P. van Duijvendijk, I. H. J. T. de Hingh, J. W. A. Leijtens, C. Sietses, E. J. Spillenaar-Bilgen, R. J. C. L. M. Vuylsteke, C. Hoff, J. W. A. Burger, W. M. U. van Grevenstein, A. Pronk, R. J. I. Bosker, H. Prins, A. B. Smits, S. Bruin, D. D. Zimmerman, L. P. S. Stassen, M. S. Dunker, M. Westerterp, P. P. Coene, J. Stoot, W. A. Bemelman, J. B. Tuynman

**Affiliations:** Department of Surgery, Academic Medical Center, University of Amsterdam, Amsterdam, The Netherlands; Department of Radiotherapy, Leiden University Medical Center, Leiden, The Netherlands; Department of Gastroenterology, Academic Medical Center, University of Amsterdam, Amsterdam, The Netherlands; Department of Gastroenterology, Antoni Van Leeuwenhoek, Amsterdam, The Netherlands; Department of Pathology, Antoni Van Leeuwenhoek, Amsterdam, The Netherlands; Department of Pathology, VU University Medical Center, Amsterdam, The Netherlands; Department of Pathology, RadboudUMC, Nijmegen, The Netherlands; Department of Medical Oncology, Academic Medical Center, University of Amsterdam, Amsterdam, The Netherlands; Clinical Research Unit, Academic Medical Center, University of Amsterdam, Amsterdam, The Netherlands; Department of Surgery, RadboudUMC, Nijmegen, The Netherlands; Department of Surgery, Antoni van Leeuwenhoek, Amsterdam, The Netherlands; Department of Surgery, IJselland Hospital, Capelle aan de Ijssel, The Netherlands; Department of Surgery, Oxford University Hospital, Oxford, UK; Department of surgery, Tergooi Hospital, Hilversum, The Netherlands; Department of surgery, Onze Lieve Vrouwe Gasthuis, Amsterdam, The Netherlands; Department of surgery, Isala Hospital, Zwolle, The Netherlands; Department of surgery, Flevo Hospital, Almere, The Netherlands; Department of surgery, Gelre Hospital, Apeldoorn, The Netherlands; Department of Surgery, Catharina Hospital, Eindhoven, The Netherlands; Department of Surgery, Laurentius Hospital, Roermond, The Netherlands; Department of Surgery, Gelderse Vallei Hospital, Ede, The Netherlands; Department of Surgery, Rijnstate Hospital, Arnhem, The Netherlands; Department of Surgery, Spaarne Gasthuis, Haarlem, The Netherlands; Department of Surgery, Medisch Centrum Leewarden, Leeuwarden, The Netherlands; Department of Surgery, Erasmus Medical Center, Rotterdam, The Netherlands; Department of Surgery, University Medical Center, Utrecht, The Netherlands; Department of Surgery, Diaconessenziekehuis, Utrecht, The Netherlands; Department of Surgery, Deventer Hospital, Deventer, The Netherlands; Department of Surgery, Jeroen Bosch Hospital, Den Bosch, The Netherlands; Department of Surgery, Sint. Antonius Hospital, Nieuwegein, The Netherlands; Department of Surgery, Slotervaart Hospital, Amsterdam, The Netherlands; Department of Surgery, Elisabeth-TweeSteden Hospital, Tilburg, The Netherlands; Department of Surgery, MUMC, Maastricht, The Netherlands; Department of Surgery, Noordwest Ziekenhuisgroep, Alkmaar, The Netherlands; Department of Surgery, Medical Center Haaglanden, The Hague, The Netherlands; Department of Surgery, Maasstad Hospital, Rotterdam, The Netherlands; Department of Surgery, Zuyderland Hospital, Sittard, The Netherlands; Department of Surgery, VU University Medical Center, De Boelelaan 1117, 1081 HZ Amsterdam, The Netherlands

**Keywords:** Rectal cancer, Organ preserving, TEM, Adjuvant chemoradiotherapy

## Abstract

**Background:**

Rectal cancer surgery is accompanied with high morbidity and poor long term functional outcome. Screening programs have shown a shift towards more early staged cancers. Patients with early rectal cancer can potentially benefit significantly from rectal preserving therapy. For the earliest stage cancers, local excision is sufficient when the risk of lymph node disease and subsequent recurrence is below 5 %. However, the majority of early cancers are associated with an intermediate risk of lymph node involvement (5–20 %) suggesting that local excision alone is not sufficient, while completion radical surgery, which is currently standard of care, could be a substantial overtreatment for this group of patients.

**Methods/Study design:**

In this multicentre randomised trial, patients with an intermediate risk T1-2 rectal cancer, that has been locally excised using an endoluminal technique, will be randomized between adjuvant chemo-radiotherapylimited to the mesorectum and standard completion total mesorectal excision (TME). To strictly monitor the risk of locoregional recurrence in the experimental arm and enable early salvage surgery, there will be additional follow up with frequent MRI and endoscopy. The primary outcome of the study is three-year local recurrence rate. Secondary outcomes are morbidity, disease free and overall survival, stoma rate, functional outcomes, health related quality of life and costs. The design is a non inferiority study with a total sample size of 302 patients.

**Discussion:**

The results of the TESAR trial will potentially demonstrate that adjuvant chemoradiotherapy is an oncological safe treatment option in patients who are confronted with the difficult clinical dilemma of a radically removed intermediate risk early rectal cancer by polypectomy or transanal surgery that is conventionally treated with subsequent radical surgery. Preserving the rectum using adjuvant radiotherapy is expected to significantly improve morbidity, function and quality of life if compared to completion TME surgery.

**Trial registration:**

NCT02371304, registration date: February 2015

## Background

The introduction of population based screening programs for colorectal cancer is expected to cause a shift towards more early stage carcinoma’s, as shown in the United Kingdom [1]. Radical rectal surgery (i.e. low anterior resection (LAR) or abdominoperineal resection (APR)) is accompanied with high operative morbidity of 36 % and is associated with a significant negative impact on functional outcome and quality of life [2–4]. More than 50 % of patients experience some form of faecal incontinence with a negative impact on quality of life. Urinary incontinence or retention and sexual dysfunction are common [2, 5–7]. Furthermore, patients after total mesorectal excision (TME) are confronted with stoma related difficulties, morbidity and subsequent hazards from stoma reversal in those with protected low anastomoses. In the Dutch TME-trial, 19 % of patients did not have a reversal of a temporary stoma after LAR and the overall long term or permanent stoma rate was 40 % [8]. After APR, up to 40 % of patients experience perineal wound complications. Long-term discomfort after APR is related to stoma and stoma appliance-related complications, occurring in up to 66 % [9]. The disadvantages of radical surgery have been acceptable in the pursuit of oncological control. However, early stage cancer might be amenable to cure by local excision with avoidance of radical surgery with its negative impact in a significant proportion of patients [10]. Local excision alone has only been considered oncological safe for low risk T1 rectal cancer, which may be defined as well/moderately differentiated without lymphatic or vascular invasion and excised with at least 1 mm margin. In case of any unfavourable histological characteristic, there is a substantial increase in the risk of lymph node metastases with impaired oncological outcome after local excision alone, requiring completion total mesorectal excision (TME) [11]. Histological characteristics which are associated with increased risk of local recurrence are: poor differentiation, deep submucosal infiltration, lymphatic or vascular invasion, SM3 and tumour size > 5 cm for pT1 or > 3 cm for pT2 [11, 12].

Early rectal cancer (T1 and T2) with intermediate risk for recurrence make up to 75 % of locally excised rectal cancer, and present a management dilemma for patient, surgeon and oncologist. These patients could be treated with additional chemo-radiotherapy, thereby preserving their rectum which likely has a positive impact on function and quality of life (HRQoL). Additional chemo-radiotherapy in the intermediate risk group has a potential to decrease the risk of local recurrence by sterilizing mesorectal lymph nodes in the vicinity of the excised tumour that may harbour (micro)metastasis. Current evidence on rectal preserving treatment options after local excision is limited, because of small sized retrospective studies with several methodological problems. Characteristics of included patients, surgical technique of local excision, and reported local recurrence rates and survival after adjuvant chemoradiotherapy for pT1-T2 carcinomas are highly variable [13–18]. We conducted a systematic review of all these studies, showing poor quality of the selected studies with lack of randomised data (Borstlap et al.). A relatively high pooled weighted average of local recurrence rate of 14 % after adjuvant radiotherapy was found, probably related to inclusion criteria that were not confined to an intermediate risk group. Only two of the 14 included studies in the review consisted of a patient population that all had clear resection margins [19, 20]. Their pooled average of 6 % recurrence after adjuvant chemoradiotherapy therapy is low, but the sample size (*n* = 27) was too small to draw any conclusion. Using strict patient selection based on MRI imaging to exclude patients with suspicious lymph nodes and pathological assessment of the excised tumour, adjuvant (chemo)radiotherapy is potentially a valid option as curative completion treatment for radically excised pT1 and small pT2. Additionally, improvements in MRI and advanced endoscopic imaging will enable careful follow-up and early detection of locoregional recurrent disease, allowing for early salvage surgery that offers acceptable oncological outcome [21].

Therefore, there is an urgent need to define a new treatment approach for patients who are confronted with invasive growth in a locally excised rectal lesion with intermediate risk of lymph node metastases. Such an approach should result in an optimal balance between treatment related morbidity and oncological control for these early stage carcinomas. A multicentre randomised controlled trial with strict inclusion criteria based on tumour characteristics and an intensive follow-up schedule in the rectal preserving treatment arm could provide the much needed evidence on oncologic outcome after adjuvant chemoradiotherapy for locally excised rectal cancer compared to standard completion TME.

## Methods/Design

The main objective of this multicentre randomised controlled trial is to determine the optimal treatment strategy for patients with a locally excised rectal lesion revealing an early stage rectal cancer with post excision pathology predicting intermediate (5–20 %) risk of recurrence. The patients will be randomised, after local excision, between either adjuvant chemoradiotherapy or standard completion TME surgery.

The TESAR trial hypothesizes that local excision combined with adjuvant chemoradiotherapy and close-surveillance with the possibility of early salvage surgery is non-inferior to completion TME in terms of local recurrence and superior in terms of treatment related morbidity, functional outcome, and HRQoL.

All pathology specimens of potentially eligible patients will be centrally revised by one pathologist in order to exclude inter-observer bias. The patient who meets all the inclusion criteria without any of the exclusion criteria will be given information about the proposed trial. The patient will have at least three days to decide upon study participation. Randomization will be performed by a central automated randomization system using the trial website, with stratification for age (75- and 75+), ASA (I and II+) classification, initial treatment (full thickness excision and endoscopic excision) and tumour classification (high risk T1 and low risk T2). The treatment, either adjuvant chemo-radiotherapy or completion TME surgery, should start within 4–8 weeks from randomisation. Randomisation will be performed in random blocks of sizes and 4.

The primary aim of the TESAR-Trial:

- To compare rectal preserving therapy (local excision followed by adjuvant chemoradiotherapy) with completion TME surgery in terms of 3-year locoregional recurrence rate.

Secondary aims of the study are:

- To compare treatment related morbidity between the study arms, both short and long-term.
- To determine 3-year and 5-year disease free survival and overall survival after both treatment strategies.
- To determine stoma-free survival at one, three and five years for both groups of patients.
- To investigate the impact of organ preserving therapy on HRQol and functional outcomes compared to radical surgery.
- Determining cost-effectiveness of rectal preserving therapy with intensified follow-up using MRI and endoscopy.

### Study procedures

**Fig. 1:**
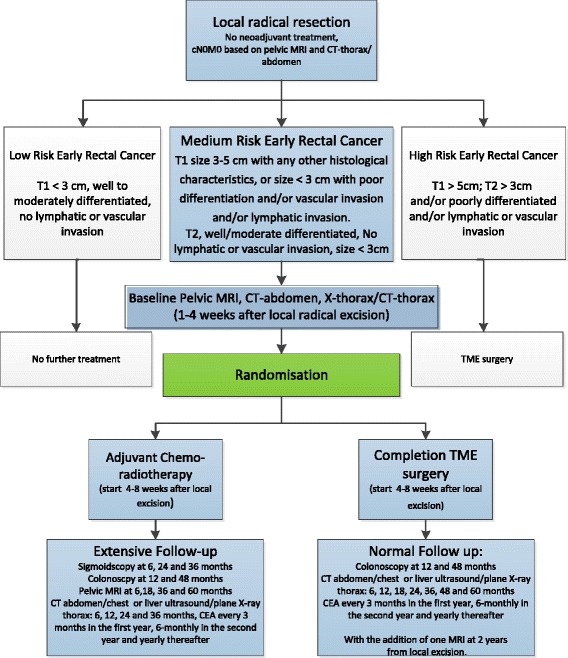
Study flowchart

Since the control arm consists of completion TME which will remove the mesorectum, the investigational arm consists of chemoradiotherapy which is primarily targeted at the mesorectum without expansion to pelvic sidewall and lymph nodes along the iliac vessels. This is based on the hypothesis that potential lymph node metastases are located close to the primary tumour site in these early stage cancers. Adapting radiation fields that are normally used in more advanced rectal cancer limits toxicity based on a patient tailored approach according to estimated recurrence risks. Patients will receive 25×1.8 Gray, 5 days a week, combined with Capecitabine 825 mg/m2 twice a day, which will not be continued during the weekends. The treatment in both study arms should start between 4-8 weeks after local excision. Patients treated with adjuvant chemoradiotherapy in the experimental arm will receive an intensified follow-up schedule with four extra pelvic MRI’s and three extra sigmoidoscopies in addition to routine follow-up according to the Dutch colorectal cancer guideline, while control patients undergo routine follow-up with one additional pelvic MRI at 24 months (see Fig. 1).

### Patient population

**Fig. 2:**
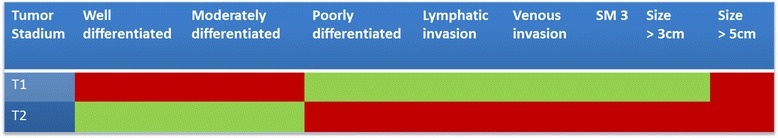
Inclusion characteristics

Patients who are diagnosed with early rectal cancer that was locally excised with tumour free margins (≥1 mm) and a carcinoma within 10 cm from the anal verge are informed about the TESAR trial if histopathology reveals the following characteristics: pT1 with a diameter of 3–5 cm, or pT1 with a maximum diameter of 3 cm and at least poor differentiation and/or lymphatic and/or venous invasion and/or SM3 invasion depth, or a pT2, maximum size 3 cm, well/moderately differentiated and without lymphatic or venous invasion (Fig. 2). Any endoluminal technique for local excision may be included (TEM, TAMIS, TSPM, EMR, ESD or polypectomy). A central review of the pathology will be performed in order to verify the histological inclusion criteria. Furthermore, clinical nodal status should be N0 by pelvic MRI. In order to prevent overstaging by post-excision MRI in these early rectal cancers, mesorectal and extra-mesorectal lymph nodes with a size smaller than 10 mm will be interpreted as benign independent of their morphologic features. Other inclusion criteria are absence of distant metastasis by CT thorax/abdomen, age > 18 years, life expectancy of at least 12 months, WHO performance status 0–2, and written informed consent.

Patients are excluded from the study or registry in case of a proximal rectal cancer (>10 cm from the anal verge or requiring partial mesorectal excision), not fulfilling pathology or imaging inclusion criteria, previous pelvic irradiation, concomitant malignancies within past 5 years, or if patients are unfit for subsequent chemoradiotherapy or surgery.

Early rectal carcinoma’s with pathological features showing low risk T1 (<5 cm, well differentiated, no lymphatic or vascular invasion) or high risk T2 (>3 cm and/or poorly differentiated and/or lymphatic or vascular invasion) will be asked for registration only and will be asked to sign informed consent to collect to use the paraffin specimen for translational studies.

### Outcome parameters

Loco-regional recurrence, morbidity, disease free survival, stoma free survival and overall survival will be assessed by regular follow-up at 3, 6, 9, 12, 18, 24, 36, 48 and 60 months post-operatively. Morbidity will be assessed using the Comprehensive Complication Index (CCI) for the patients that undergo TME surgery and the NCI CTCAE Toxicity Criteria (v4) for patients in the adjuvant chemoradiotherapy arm [22]. Health related quality of life and functional outcomes will be measured using the EQ-5D, EORTC QLQ C29 & C30 and the LARS score. Possible advantage of the new rectal preserving treatment in cost per quality of life adjusted life years using the EQ5D score will be analysed. The total costs will be assessed by summing the procedure related costs, in hospital stay costs, re-intervention and morbidity related costs and time to return to work will be calculated in loss of work days, which can be converted to costs.

### Follow-up

**Fig. 3:**
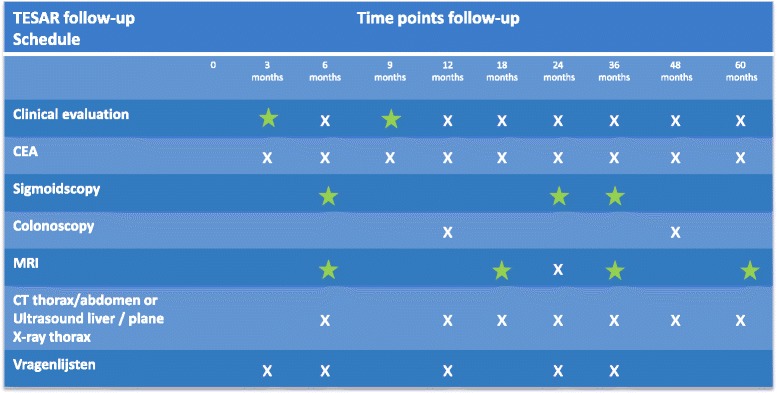
Follow-up schedule TESAR trial. Green = extra follow-up moments for included patients in the rectal preserving group. The white x’s are the follow-up moments according to national guideline. The blue star resembles the extra MRI in the control group

Patients in the intervention arm will receive an intensified follow-up schedule to facilitate an early salvage procedure in case of recurrence. Patients will have an additional clinical evaluation including digital rectal examination at 3 and 9 months and a pelvic MRI will be performed at 6,18,36 and 60 months. Besides routine colonoscopy at 12 and 48 months according to the national guideline, three additional sigmoidoscopies are performed at 6, 24 and 36 months. Patients in the control arm will receive one additional MRI, 2 years after local excision. The follow-up schedule is presented in Fig. 3.

### Sample size

The trial is designed as a non-inferiority trial. The expected percentage of patients who are free of local recurrence after a three-year follow-up is 98 % in the control group and 96 % in the study group. The difference in percentage of recurrence free patients between standard treatment and experimental treatment may not be larger than 7 %. Hence, the experimental treatment is non-inferior if at least 91 % of patients is free of local recurrence. This 91 % is seen as a worst case scenario when adjuvant treatment has no influence on local recurrence. Hypothesizing that the difference in percentage of patients with local recurrence may not be larger than 7 %, with a one sided alfa, an 80 % power and a drop-out of 5 % a sample size of 302 patients (151 per group) is needed.

### Data analysis

All analyses will be on an intention-to-treat basis [23]. Patients will be analysed as they are randomized irrespective of the treatment actually received. The intention-to-treat population will include all patients who have given their informed consent and for whom there is confirmation of successful allocation of a randomisation number. To substantiate the interpretation of the intention-to-treat analysis a per protocol analysis will also be performed.

Statistical analyses will be performed using SPSS software for Windows version 23 or higher. The one-sided 95 % confidence interval for the between-group difference in locoregional recurrence corresponds to the lower limit of the two-sided 90 % confidence interval for this difference (experimental minus standard). The organ preserving treatment group (intervention) is considered to be non-inferior to the standard treatment group if the one-sided 95 % confidence for the difference in loco-regional recurrence excludes a difference of minus 7 percentage points or farther away from zero in the same direction. For the secondary outcomes as disease free survival and overall survival two-sided 95 % confidence intervals will be calculated. Appropriate summary descriptive statistics will be determined for all secondary endpoints at each visit using raw scores. To assess morbidity the continuous scale of the Comprehensive Complication Index [22] and the categorical Clavien-Dindo classification will be used, for which appropriate statistical tests will be undertaken (the Mann Whitney *U* test, in case of non-normal distribution, and the Chi squared test respectively). For continuous secondary endpoints, either analysis of variance or analysis of covariance models will be used and where necessary, the repeated measures procedure will be implemented. Quality of life data (e.g. EORTC-C30, EORTC-CR29, LARS and EQ-5D) will be graphically represented across all time points and analysed using a repeated measures analysis of variance. All tests based on proportions will be analysed using a logistic regression model with treatment as a factor and, where appropriate, other specified covariates. Time-to-event will be analysed with use of Kaplan-Meier Survival analysis and compared using the log-rank test. The secondary efficacy analysis will be based on the Full Analysis Set (FAS) and the per protocol populations. Significance level is set at an alpha of 0.05 and no adjustment will be made for testing multiple secondary outcomes. Some significant findings are expected to occur by chance so undue consideration will not be given to any particular significant difference. Moreover, interpretation of the results will be based on patterns of differences and in conjunction with the results of the primary analyses.

### Cost analysis

Unit costing of distinct health care resources will be in accordance with national guidelines (<http://docplayer.nl/6361834-Handleiding-voor-kostenonderzoek-methoden-en-standaard-kostprijzen-voor-economische-evaluaties-in-de-gezondheidszorg.html>). Cost data will be derived as the product sum of health care volume data and their respective unit cost. Observed health utilities based on the EQ-5D health status profiles will be linked to the lengths of the periods in between measurements to derive QALYs. Incremental cost-effectiveness and cost-utility analyses will be performed to calculate the extra cost per additional patient free of local recurrence and the extra costs per additional quality adjusted life year respectively. Differences between groups will be assessed by calculating 95 % confidence intervals for the mean differences after non-parametric bootstrapping, drawing at least 1000 samples of the same size as the original sample separately for each group and with replacement. A cost-effectiveness acceptability curve will be drawn to show the probability of adjuvant chemoradiotherapy being cost- effective at willingness-to-pay values up to €80,000 per QALY. Single and multi-way sensitivity analyses will be performed to study the robustness of these findings to plausible changes in key unit costs and to alternative health utility scoring algorithms [24]. Efficacy and cost data will be discounted to account for time preference.

## Discussion

The TESAR-Trial is the first multicentre randomised trial in which patients with a pT1-T2 rectal carcinoma will be randomised after local excision between rectal preserving adjuvant chemoradiotherapy and completion TME. In this non-inferiority trial we hypothesize that rectal preserving therapy for early rectal cancer has similar oncological outcome with significant improved morbidity, function and quality of life compared to conventional radical surgery. The TESAR-Trial is part of a national treatment consortium of multicentre studies founded to create sufficient evidence on the best treatment strategies for early rectal cancer.

The TREC trial from the UK (TEM and Radiotherapy in Early Rectal Cancer) included patients with clinically staged early (cT1-2N0M0) rectal cancer and randomised between radical TME surgery and short course preoperative radiotherapy followed by TEM [REF]. The CARTS phase II Dutch multicentre study prospectively evaluated outcome after chemo-radiotherapy followed by TEM for clinical T1-3N0M0 rectal cancer [25]. Recently it was proposed to combine both initiatives in a new protocol: the STARTREC trial. This study will be conducted parallel to the TESAR trial in the Netherlands. The STARTREC study will be randomizing between intentional organ preserving therapy and radical TME surgery. The STARTREC will include patients based on imaging with the rectal cancer is still in situ. Patients with small tumours staged as T1N0, less then 3 cm can still be treated with initial local excision according to the national guideline. If histopathology shows intermediate risk features, they are eligible for the TESAR trial. Moreover, a large proportion (up to 40 %) of rectal cancer is diagnosed and discussed at the rectal cancer Multi-Disciplinary Team meeting (MDT) after the patient has had an endoluminal local- excision of a lesion that shows infiltrative growth at pathological examination. These excisions might have been polypectomies within the national screening program or an excision of a clinically diagnosed adenoma by for example TEM or EMR [11]. This subgroup of patients can therefore not be included in the STARTREC trial, but will be included in the TESAR. Both TESAR and STARTREC use the same patient reported outcome measurements allowing comparative combined analysis. Together, potentially all patients with early rectal cancer can be included and results from the randomised trials will be powerful to answer which is the best treatment strategy for patients with early rectal cancer.

Current literature on adjuvant chemoradiotherapy after local excision consists of small cohort series which show substantial variability in recurrence rates, ranging between 6 and 43 %, [15–19, 26–34]. Patient selection in these series was often unclear, but often chemoradiotherapy was chosen in patients refusing further surgery or being unfit for completion TME despite they had an incomplete resection or high risk T2 rectal cancer. This explains the relatively high recurrence rates in these series. Furthermore, a variety in adjuvant radiotherapy regimens was used, and none of the studies reported a strict follow-up schedule including digital rectal examination, MRI and endoscopy to allow for early salvage treatment.

Within the TESAR trial, strict pathological selection criteria based on differentiation grade, lymphatic- or vascular invasion and size of the tumour are used in order to include only the intermediate risk group and to exclude patients at high risk of local recurrence. Therefore we expect that the risk of recurrence after adjuvant chemoradiotherapy following local excision will be lower than reported so far. Treatment related morbidity from the chemoradiotherapy is expected to be lower than earlier reported [25]. This is related to the radiation field which is limited to the mesorectum and a lower total dose of 45 Gy than generally applied in more advanced rectal cancer. Additionally, concomitant chemotherapy is only given on weekdays. This less intensive chemoradiotherapy schedule is considered to be oncologically save because only radically removed early rectal carcinomas are included. Nevertheless, patients in the rectal preserving group of the TESAR are expected to have a slightly higher risk of recurrence than the control group. However, this increase is accepted due to the close standardised follow up schedule which allows for early salvage of recurrence. To ensure adequate patient inclusion with the anticipated intermediate risk of recurrence, all specimens of the locally excised tumours are centrally reviewed before a patient can be included in the trial.

In conclusion, due to a shift towards more early staged carcinomas shift and high morbidity of TME surgery, there is an increasing need for less invasive treatment approaches with acceptable oncological outcome. After local excision has revealed a high risk T1 or low risk T2 carcinoma, adjuvant chemoradiotherapy could be an oncological safe alternative for radical surgery, with potential improvements in treatment related morbidity, functional outcome and quality of life. This will prospectively be evaluated in the randomised multicentre TESAR trial.

## Abbreviations

APR, abdominoperineal resection; CCI, comprehensive complication index; DSMB, data safety monitoring board; EMR, endoscopic mucosal resection; ESD, endoscopic submucosal dissection; FAS, full analysis set (FAS); HRQoL, health related quality of life; KWF, Dutch Cancer Society; LAR, low anterior resection; METC, Medical research ethics committee (MREC) (in Dutch, Medisch Ethische ToetsingsCommissie; QALY, quality adjusted life years; TAMIS, transanal minimally invasive surgery; TEM, transanal endoscopic microsugery; TESAR, rectal preserving treatment for early rectal cancer. A multi-centred randomised trial of radical surgery versus adjuvant chemoradiotherapy after local excision for early rectal cancer; TME, total mesorectal excision; TSPM, transanal single-port microsurgery; WMO, WMO: Medical Research Involving Human Subjects Act (in Dutch: Wet Medisch-wetenschappelijk Onderzoek met mensen
